# C/EBPδ Deficiency Sensitizes Mice to Ionizing Radiation-Induced Hematopoietic and Intestinal Injury

**DOI:** 10.1371/journal.pone.0094967

**Published:** 2014-04-18

**Authors:** Snehalata A. Pawar, Lijian Shao, Jianhui Chang, Wenze Wang, Rupak Pathak, Xiaoyan Zhu, Junru Wang, Howard Hendrickson, Marjan Boerma, Esta Sterneck, Daohong Zhou, Martin Hauer-Jensen

**Affiliations:** 1 Division of Radiation Health, Department of Pharmaceutical Sciences, University of Arkansas for Medical Sciences, Little Rock, Arkansas, United States of America; 2 Department of Pharmaceutical Sciences, University of Arkansas for Medical Sciences, Little Rock, Arkansas, United States of America; 3 Center for Cancer Research, National Cancer Institute, Frederick, Maryland, United States of America; 4 Surgical Service, Central Arkansas Veterans Healthcare System, Little Rock, Arkansas, United States of America; National Cancer Institute, United States of America

## Abstract

Knowledge of the mechanisms involved in the radiation response is critical for developing interventions to mitigate radiation-induced injury to normal tissues. Exposure to radiation leads to increased oxidative stress, DNA-damage, genomic instability and inflammation. The transcription factor CCAAT/enhancer binding protein delta (*Cebpd*; C/EBPδ is implicated in regulation of these same processes, but its role in radiation response is not known. We investigated the role of C/EBPδ in radiation-induced hematopoietic and intestinal injury using a *Cebpd* knockout mouse model. *Cebpd*−/− mice showed increased lethality at 7.4 and 8.5 Gy total-body irradiation (TBI), compared to *Cebpd*+/+ mice. Two weeks after a 6 Gy dose of TBI, *Cebpd*−/− mice showed decreased recovery of white blood cells, neutrophils, platelets, myeloid cells and bone marrow mononuclear cells, decreased colony-forming ability of bone marrow progenitor cells, and increased apoptosis of hematopoietic progenitor and stem cells compared to *Cebpd+/+* controls. *Cebpd*−/− mice exhibited a significant dose-dependent decrease in intestinal crypt survival and in plasma citrulline levels compared to *Cebpd+/+* mice after exposure to radiation. This was accompanied by significantly decreased expression of γ-H2AX in *Cebpd*−/− intestinal crypts and villi at 1 h post-TBI, increased mitotic index at 24 h post-TBI, and increase in apoptosis in intestinal crypts and stromal cells of *Cebpd*−/− compared to *Cebpd+/+* mice at 4 h post-irradiation. This study uncovers a novel biological function for C/EBPδ in promoting the response to radiation-induced DNA-damage and in protecting hematopoietic and intestinal tissues from radiation-induced injury.

## Introduction

Ionizing radiation (IR) is commonly used to treat a wide variety of cancers. Despite improvements in radiation technology, the delivery of radiotherapy to a tumor unavoidably affects the normal tissues surrounding it. Normal tissue toxicity often is a dose-limiting factor, as the therapeutic ratio of radiotherapy is dictated by a balance between normal tissue toxicity and tumor control [1], [2]. Normal tissue injury is also a major side-effect of exposure to whole-body radiation, either in a clinical setting or during a nuclear accident. Normal tissue radiation toxicity mainly affects rapidly renewing cell systems, such as bone marrow and gastrointestinal (GI)-tract mucosa. There is a need for more effective agents to counteract IR-induced normal tissue injury [1], [2].

IR-induced acute bone marrow injury leads to a decrease in the number of circulating blood cells, and most of the post-radiation hematopoietic morbidity and mortality is attributed to infection and hemorrhage due to leukopenia and thrombocytopenia [2]. Induction of apoptosis in the rapidly proliferating hematopoietic progenitor cells (HPCs) and, to a lesser degree, the relatively quiescent hematopoietic stem cells (HSCs) leads to acute myelosuppression [3], [4]. In addition to acute bone marrow injury, long-term bone marrow injury also results from acute radiation exposure and manifests as a decrease in HSC reserves and impairment in HSC self-renewal [3]–[8].

Radiation-induced GI damage is another complication of IR exposure and is characterized by a loss in the progenitor cell compartment (impaired epithelial renewal, villus atrophy), microvascular endothelial cell death, and mucosal inflammation [9]. Damage to stem cells plays a critical role in this process [9]. Although bone marrow aplasia in patients accidentally exposed to a lethal radiation dose can be rescued by bone marrow or stem cell transplantation, or by cytokine therapy such as G-CSF or GM-CSF [10]–[12], there is currently no medical countermeasure against GI injury.

Studies in knockout mouse models of p53, targets of p53, NF-κB, and other genes led to identification of some of the molecular mechanisms that drive cellular, tissue, and organismal responses to radiation [13]–[19]. However, additional mechanisms that contribute to the radiation response remain undefined, and investigations in this direction will aid in the development of novel target-specific interventions to protect normal tissues from radiation injury thus improving the efficacy of cancer radiotherapy [20].

Exposure to IR leads to increased oxidative stress, DNA-damage, genomic instability and an increased inflammatory response; the transcription factor CCAAT enhancer binding protein delta (C/EBPδ) is implicated in regulation of these same processes [21]–[24], [25], [27], but its role in radiation response has not been investigated. This study examines the role of C/EBPδ in the response to IR, using a *Cebpd* -knockout mouse model. Although all C/EBP family members share the same DNA-binding specificity, they have highly diverse tissue-specific functions [25]. *Cebpd−/−* mice are viable with no overt phenotype [26]. Work by several groups has implicated C/EBPδ in the regulation of target genes of diverse biological functions that include acute-phase response, growth arrest, apoptosis, differentiation, stem cell self-renewal, and tumor suppression [25], [27]–[32]. C/EBPδ alleviates oxidative stress via the transcriptional regulation of superoxide dismutase1 (SOD1) [21], and is implicated in DNA damage repair via its interactions with the Fanconi anemia protein D2 [22]. We previously demonstrated that loss of C/EBPδ promotes genomic instability [23], in part because of cyclin D1 overexpression [24].

We report here that lack of C/EBPδ is associated with an impaired DNA-damage response, increased apoptosis of the HPCs, HSCs, and intestinal stem and villi stromal cells, followed by reduced survival in response to total-body irradiation (TBI). This study demonstrates that C/EBPδ plays an important role in the response of normal tissues to radiation and recovery from radiation injury.

## Materials and Methods

### Ethics Statement

This study was carried out in strict accordance with the recommendations in the Guide for the Care and Use of Laboratory Animals of the National Institutes of Health. and approved by the Institutional Animal Care and Use Committee of the University of Arkansas for Medical Sciences.

### Animals

*Cebpd*-heterozygous breeder mice were as described [26] and were backcrossed for more than 20 generations to the C57BL/6 strain background. Genotyping was done as described previously [26]. In all the studies, 10–12 weeks old subjects were derived from heterozygous mating pairs and litter mate controls were used whenever possible. The animals were housed in Division of Laboratory Medicine (DLAM, University of Arkansas for Medical Sciences, Little Rock, Arkansas) under standardized conditions with controlled temperature and humidity and a 12 h day, 12 h night light cycle. The intestine tissue harvests and bone marrow isolation procedures were performed, following intra-peritoneal injection of sodium pentobarbital (0.5 mg/10 g body weight) to minimize suffering, and the animals were euthanized by cervical dislocation.

### Irradiation of mice

TBI was administered in a Mark I irradiator (J.L. Sheperd). Dose uniformity was assessed by an independent company (Ashland Specialty Ingredients) with radiographic film and alanine tablets. Alanine tables were analyzed by the National Institute of Standards and Technology (Gaithersburg, MD) and demonstrated a dose rate of 1.14 Gy/min at 21 cm from the source. For each experiment the dose rate was corrected for decay.

### Survival studies

For studies of post-irradiation survival, *Cebpd−/−* and *Cebpd+/+* mice (n = 7–12 per genotype) were exposed to TBI doses of 7.4 and 8.5 Gy. Mice were monitored up to 30 days after TBI, and the numbers of dead or moribund mice were recorded twice daily. Animals that were moribund (more than 25% weight loss, lethargy, huddling and/or shivering, loss of appetite, hunched posture, severe diarrhea, and vocalization) were euthanized without delay as recommended by the DLAM veterinarians. Most mice in this study died without showing any of the overt clinical signs used for humane endpoints and were found dead during the daily monitoring. Kaplan-Meier survival curves were plotted and analyzed with the log-rank test.

### Isolation of blood and bone marrow cells

Mice of both genotypes were subjected to a sublethal-TBI dose of 6 Gy. On day 14 post-irradiation, following intra-peritoneal injection of sodium pentobarbital (0.5 mg/10 g body weight), peripheral blood was collected (orbital puncture method). Blood parameters were determined by a Hemavet Instrument (Drew Scientific, Inc.). Bone marrow cells (BMCs) were harvested from the tibias and femurs of *Cebpd−/−* and *Cebpd+/+* mice (n = 3–6 per genotype) followed by cervical dislocation as described previously [7], [8], [33]. BMC viability was assessed with trypan blue dye exclusion and counts were determined with a hemocytometer.

### Colony-forming unit (CFU) assay of BMCs

CFU assays were performed with MethoCult GF (M3434, Stem Cell Technologies) according to the manufacturer's instructions as described previously [8], [33]. BM-mononuclear cells (BM-MNCs; 2×10^4^) were plated in triplicate in 12-well plates and were incubated at 37°C in 5% CO_2_; colonies of burst-forming-unit–erythroid (BFU-E) and CFU–granulocyte macrophage (GM) were scored on day 7; those of CFU-granulocyte, -erythrocyte, -monocyte, and -megakaryocyte (GEMM) were scored on day 12 of the incubation. In both sets of plates, colonies consisting of ≥40 cells were enumerated by using a Carl Zeiss Invertoskop 40C microscope with a 10× objective. Colony counts were expressed as plating efficiency, which is the number of colonies per 2×10^4^ nucleated BMCs.

### Flow cytometry to determine differentiated hematopoietic lineages from peripheral blood

Peripheral blood was obtained at day 14 post-irradiation as described above. Red blood cells (RBCs) were lysed with 0.15 M NH_4_Cl, followed by blocking with staining buffer (Hank's buffered salt solution plus 2% fetal bovine serum) containing F_c_-block for 10 min before staining with antibodies. For analysis of differentiated cells, the cells were stained with PE-conjugated anti-CD11b (eBioscience) and PE-conjugated anti-Gr-1 (for myeloid cells) (eBioscience), APC-conjugated anti-Thy1.2 (for T cells) (eBioscience) or PE-conjugated anti-B220 (for B cells) (eBioscience) for 30 min on ice, followed by incubation with propidium iodide for 5 min on ice. Stained cells were washed with staining buffer and resuspended in 0.1 ml of staining buffer. For each sample, a minimum of 2×10^5^ cells were analyzed on a flow cytometer (LSR II, Becton Dickinson), and the data were analyzed with Cell Quest software (Becton Dickinson) after gating on viable cells. The percentage of B, T and myeloid cells in each mouse were calculated by multiplying the absolute numbers with the total numbers of WBCs harvested from each mouse per 0.1 ml of blood.

### Flow cytometry of HPCs and HSCs after irradiation

BMCs (2×10^6^) were blocked with Fc-block for 10 min, followed by staining with biotin-conjugated anti-CD3e (eBioscience), anti-CD45R/B220 (eBioscience), anti-Gr-1 (eBioscience), anti-CD11b (eBioscience), and anti-Ter-119 (eBioscience) antibodies for 30 min. Cells were then washed once and stained for 30 min with antibodies (1∶100 dilution) to c-Kit (CD117 2B8, fluorescein APC-conjugated; BD Pharmingen), Sca1 (Ly-6A/E, PE-conjugated; eBioscience), and streptavidin (fluorescein isothiocyanate [FITC]-conjugated; BD Pharmingen). Cells were analyzed with an LSR II flow cytometer and FlowJo software. Percentage and absolute number of HPCs (Lin^−^c-kit^+^Sca1^+^ or LKS^+^) and HSCs (Lin^−^c-kit^+^Sca1^−^ or LKS^−^) were determined by flow cytometry. The numbers of HPCs and HSCs in each mouse were calculated by multiplying the total numbers of BMCs harvested from both tibias and femurs of each mouse by the frequency of each population in BMCs.

### Apoptosis assay

BMCs from *Cebpd*−/− and *Cebpd+/+* mice were incubated with Fc-block at 4°C for 15 min to block the Fc-γ receptors and were then stained with antibodies against various cell surface markers in the dark, as described above. BMCs were stained with Annexin V according to the manufacturer's instructions, using an Annexin V-FITC kit (BD Pharmingen). The apoptotic cells in different hematopoietic cell populations were analyzed with an LSR II flow cytometer.

### Intestinal crypt colony assay and determination of plasma citrulline concentrations

Microcolony crypt survival assays were performed as described previously [34], [35]. Briefly, mice of both genotypes (n = 4–8 per group) were anesthetized with intraperitoneal injection of sodium pentobarbital (0.5 mg/10 g body weight) and sacrificed 3.5 days after TBI (0, 7.4, 8.5, and 10 Gy) followed by euthanasia by cervical dislocation. The segments of proximal jejunum were obtained, fixed, embedded (four transverse sections per specimen), cut into 3–5-µm slices, and stained with hematoxylin and eosin. Surviving crypts (those with ≥10 adjacent, chromophilic, non-Paneth cells) were counted. Four circumferences of proximal jejunum were scored per mouse, and microcolony survival was expressed as the average number of surviving crypts per circumference. The average from each mouse was considered a single value for statistical purposes.

Whole blood was collected into EDTA-coated tubes (Fisher Scientific), from irradiated animals at 3.5 days after 8.5 Gy TBI and from unirradiated mice, prior to the tissue harvest. Plasma was obtained by centrifugation (5000 *g*, 5 min, 4°C) and stored at −80°C until analyzed [36]. Plasma citrulline concentrations were determined with liquid chromatography tandem mass spectrometry as previously described [37].

### Immunohistochemistry and Terminal deoxynucleotidyl transferase dUTP nick end labeling assay

Jejunums were collected from *Cebpd*+/+ and *Cebpd*−/− mice 1 h, 4 h, 24 h, and 3.5 days post-TBI (7.4 Gy), after intra-peritoneal injection of sodium pentobarbital. The intestine tissues were fixed with methanol carnoy's fixative (methanol: chloroform: acetic acid, 6∶3∶1) for 24 h, dehydrated, were embedded in paraffin in cross-sectional orientation. For staining, sections of 5-µm thickness were cut, dewaxed, rehydrated in PBS (10 mM sodium phosphate, pH 7.4; 140 mM NaCl), and pretreated with proteinase K (20 µg/ml) for 20 min at 37°C. For control sections, blocking buffer contained blocking solution only (no primary antibody).

Sections were co-stained for terminal deoxynucleotidyl transferase dUTP nick end labeling assay (TUNEL) assays with the *In Situ* Cell Death Detection Kit (Roche Diagnostics) according to the manufacturer's protocol and with rabbit anti-γ-H2A.X (S139) (Cell Signaling Technology) at 1∶200 dilution in blocking buffer (0.5% BSA, 0.05% Tween-20, PBS). Each section was probed with a mixture of terminal deoxynucleotidyl transferase and FITC-labeled precursor in cacodylate buffer and goat anti-rabbit IgG-AlexaFluor 594 (Invitrogen) at 1∶400 dilution for 1 h at 37°C and then rinsed three times with 0.05% Tween-20 in PBS. TUNEL specificity was controlled by using cacodylate buffer instead of the mixture of terminal deoxynucleotidyl transferase and probe. After staining, sections were counterstained with 4', 6-diamidino-2-phenylindol to visualize cell nuclei and were mounted under cover slips with Prolong Antifade kit (Invitrogen).

Images for TUNEL and γ-H2AX staining were acquired with an Olympus IX-51 inverted microscope (Olympus America) with a 10× objective, equipped with Hamamatsu ORCA-ER monochrome camera (Hamamatsu Photonics K.K.). Major tissue compartments (crypts, villi and stroma) were marked manually and samples for TUNEL staining were quantified as described previously [38]. Apoptosis index was scored on a cell position basis as described previously by analysis of 50–100 half crypt sections per mouse with at least 4 mice in each group [39].

For γ-H2AX staining, the crypts and villi were marked in a total of 10 fields of view per tissue section (10× objective), and the mean intensity was measured in each compartment. The average mean intensity of the field of view per tissue section was considered as a datapoint for statistical analysis. The data are presented as mean fluorescence intensity per field in n = 4 animals per timepoint. Slidebook 4.2 software (Intelligent Imaging Innovations, Inc.) was used for image capturing and analysis.

Samples were probed with phospho-histone H3 (Ser28) (Abcam) at 1∶100 dilution in blocking buffer (2% goat serum, PBS). The phospho-histone H3 expression was detected with biotinylated anti-rat IgG (Vector Laboratories, BA-9401) at 1∶400 dilution and developed with 3, 3' diaminobenzidine tetrahydrochloride. Images for phospho-histone H3 were captured using Aperio CS bright field digital slide scanner at 40× magnification. The phospho-Histone H3 positive cells were quantified in at least 100 crypts. The average mitotic index was derived from the number of mitotic cells per crypt and calculated as a mean of 4 animals per group.

### Statistical analyses

**Figure 1:**
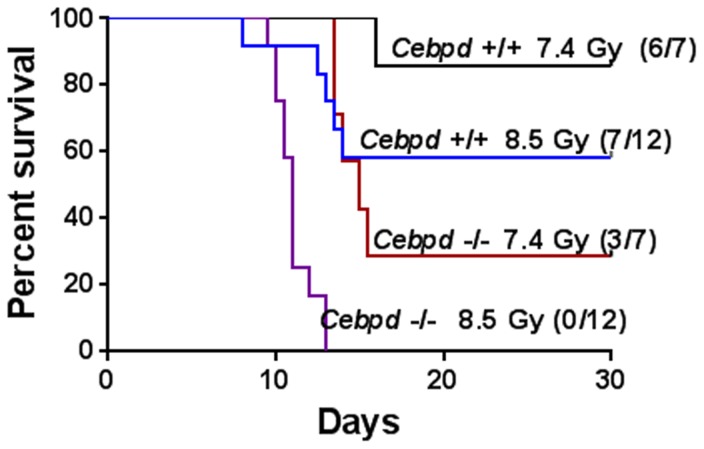
*Cebpd−/−* mice showed increased radiosensitivity to TBI. Thirty-day survival of *Cebpd−/−* mice and *Cebpd+/+* control mice exposed to 7.4 Gy (n = 7 per genotype) or 8.5 Gy (n = 12 mice per genotype) of TBI. *P = 0.02* for 7.4 Gy; *P*<0.0001 for 8.5 Gy, as calculated by Logrank (Mantel-Cox) test. The numbers in parentheses indicate the number of animals that survived.

**Figure 2:**
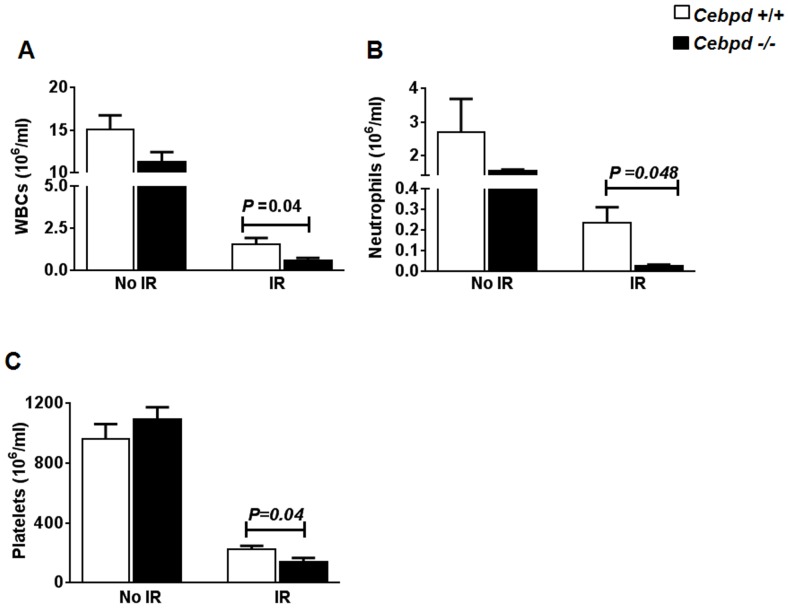
*Cebpd−/−* mice showed impaired post-irradiation recovery of WBCs, neutrophils and platelets. (A) WBCs (n = 6), (B) neutrophils (n = 3), and (C) platelets (n = 6) from unirradiated (No IR) and irradiated (IR) *Cebpd+/+* and *Cebpd*−/− mice were counted 14 days after exposure to 6 Gy TBI. All data are represented as mean ± standard error mean (SEM).

**Figure 3:**
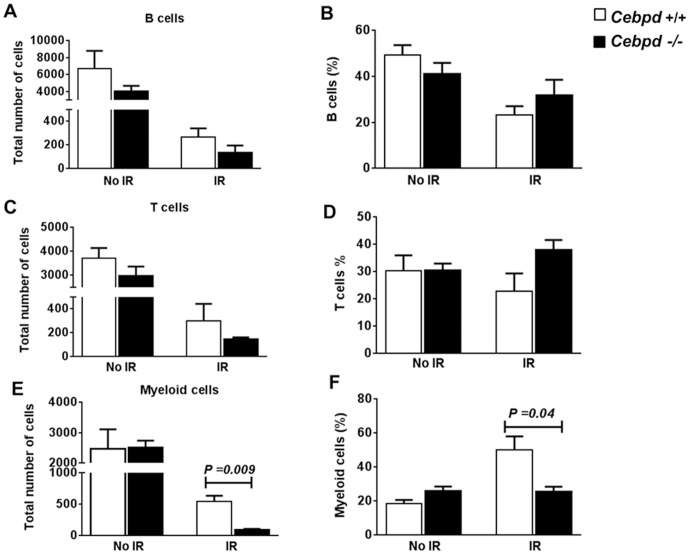
*Cebpd−/−* deficiency enhanced radiation-induced myelosuppression. Peripheral blood B cells, T cells, and myeloid cells from unirradiated (No IR) and irradiated (IR) *Cebpd+/+* and *Cebpd*−/− mice (n = 3/genotype) were enumerated 14 days after exposure to 6 Gy TBI by phenotyping (A, C, E) and expressed as percent of total WBCs (B, D, F). All data are represented as mean ± SEM.

**Figure 4:**
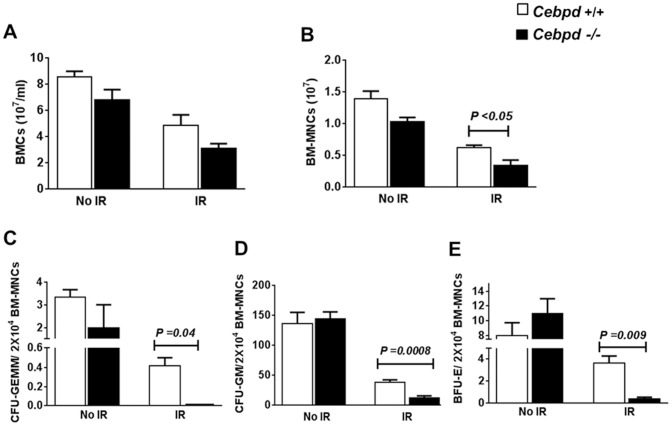
*Cebpd*-deficiency resulted in radiosensitization of bone marrow mononuclear cells and bone marrow progenitors. (A–B) BMCs and BM-MNCs were enumerated per pair of femurs and tibias per mouse from unirradiated (No IR) and irradiated (IR) *Cebpd+/+* and *Cebpd*−/− mice (n = 3/genotype) 14 days after 6 Gy TBI. (C–E) Effects of TBI are depicted for CFU-GEMM, CFU-GM, and BFU-E assays of BMC precursors from unirradiated and irradiated *Cebpd +/+* and *Cebpd*−/− mice 14 days after sublethal TBI (6 Gy). All data are represented as mean ± SEM.

**Figure 5:**
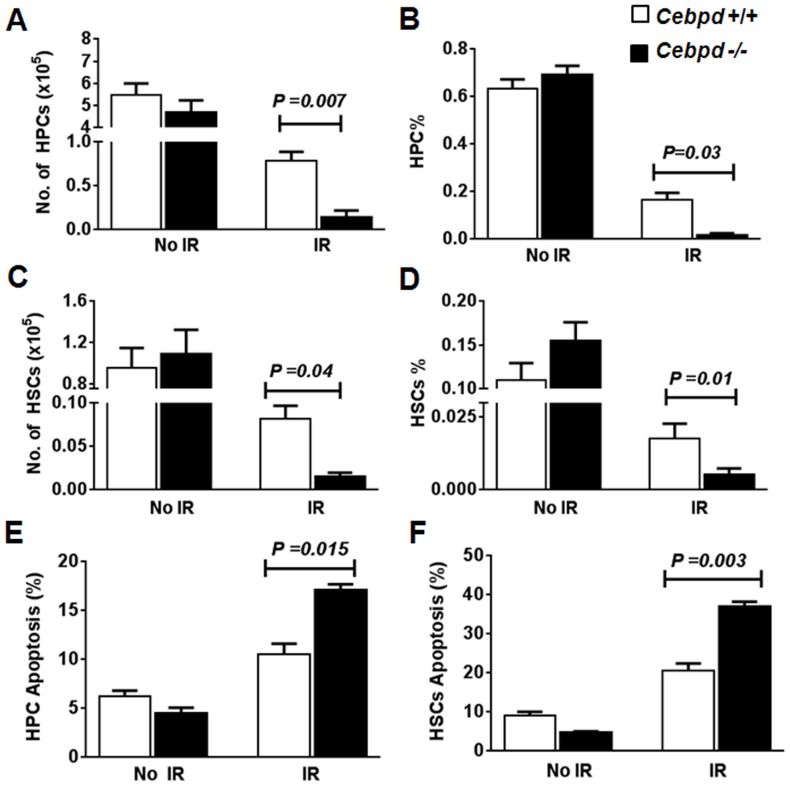
Impaired recovery of HSCs and HPCs in *Cebpd−/−* mice was due to increased apoptosis. Absolute numbers and percentages of HPCs (A, B) and HSCs (C, D) were analyzed from bone marrow of unirradiated (No IR) and irradiated (IR) *Cebpd+/+* and *Cebpd*−/− mice (n = 3/genotype) 14 days after 6- Gy TBI. Apoptosis of HPCs (E) and HSCs (F) were analyzed with Annexin V-based assays. Data are presented as an average ± SEM, n = 3 mice per group.

**Figure 6:**
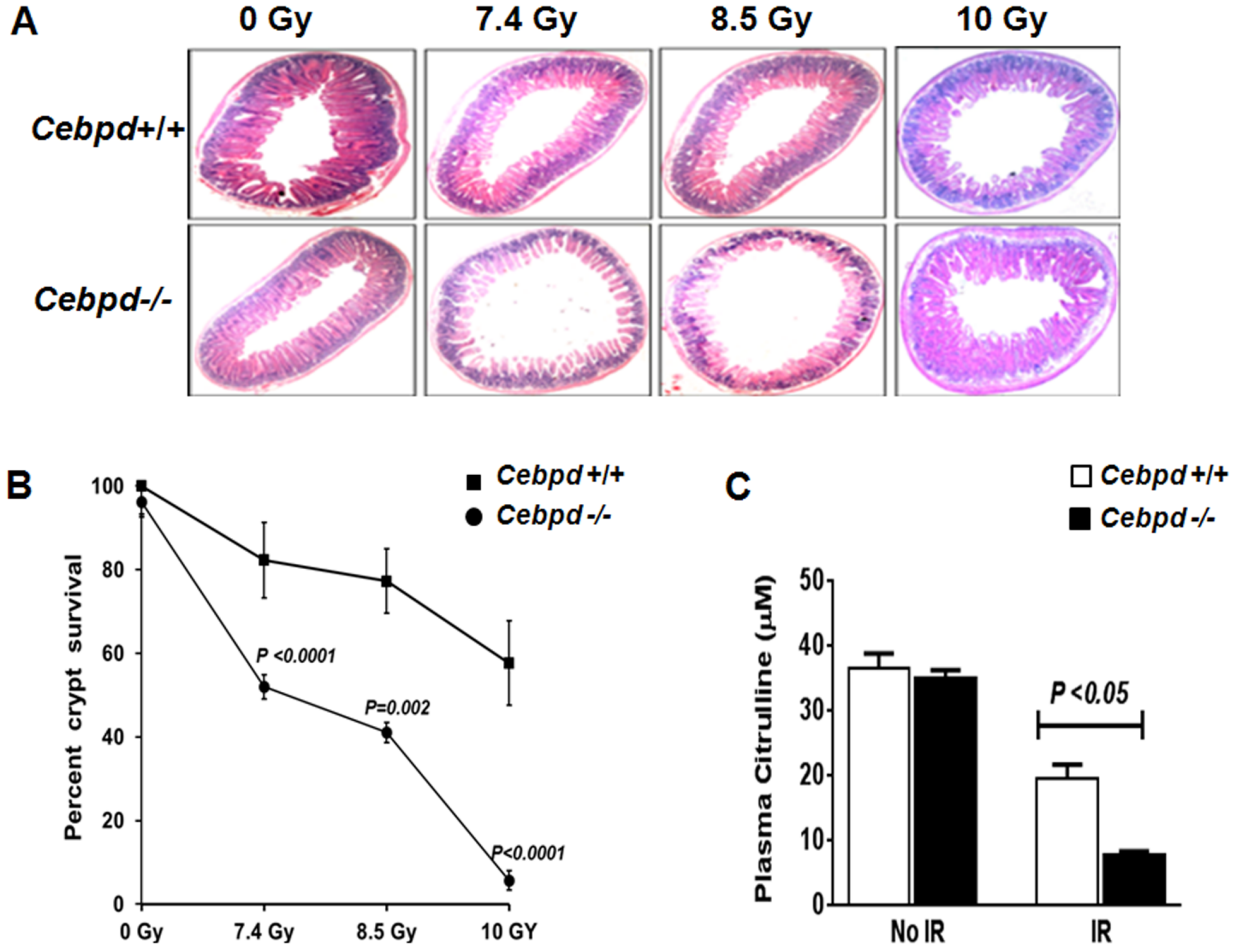
*Cebpd−/−* mice displayed increased loss of intestinal crypts in response to TBI. *Cebpd−/−* and *Cebpd+/+* mice were subjected to 0, 7.4, 8.5, and 10 Gy TBI, and tissues were obtained at day 3.5 post-irradiation. (A) Representative image of hematoxylin-eosin stained intestinal crypts in transverse sections (4X). (B) Using microcolony assays, surviving crypts from *Cebpd +/+* and *Cebpd*−/− mice were scored. (C) Plasma citrulline levels in *Cebpd−/−* and *Cebpd+/+* mice were determined before and 3.5 days after 8.5 Gy TBI. All data are presented as mean ± SEM, n = 4 mice per group. No IR: unirradiated; IR: irradiated.

**Figure 7:**
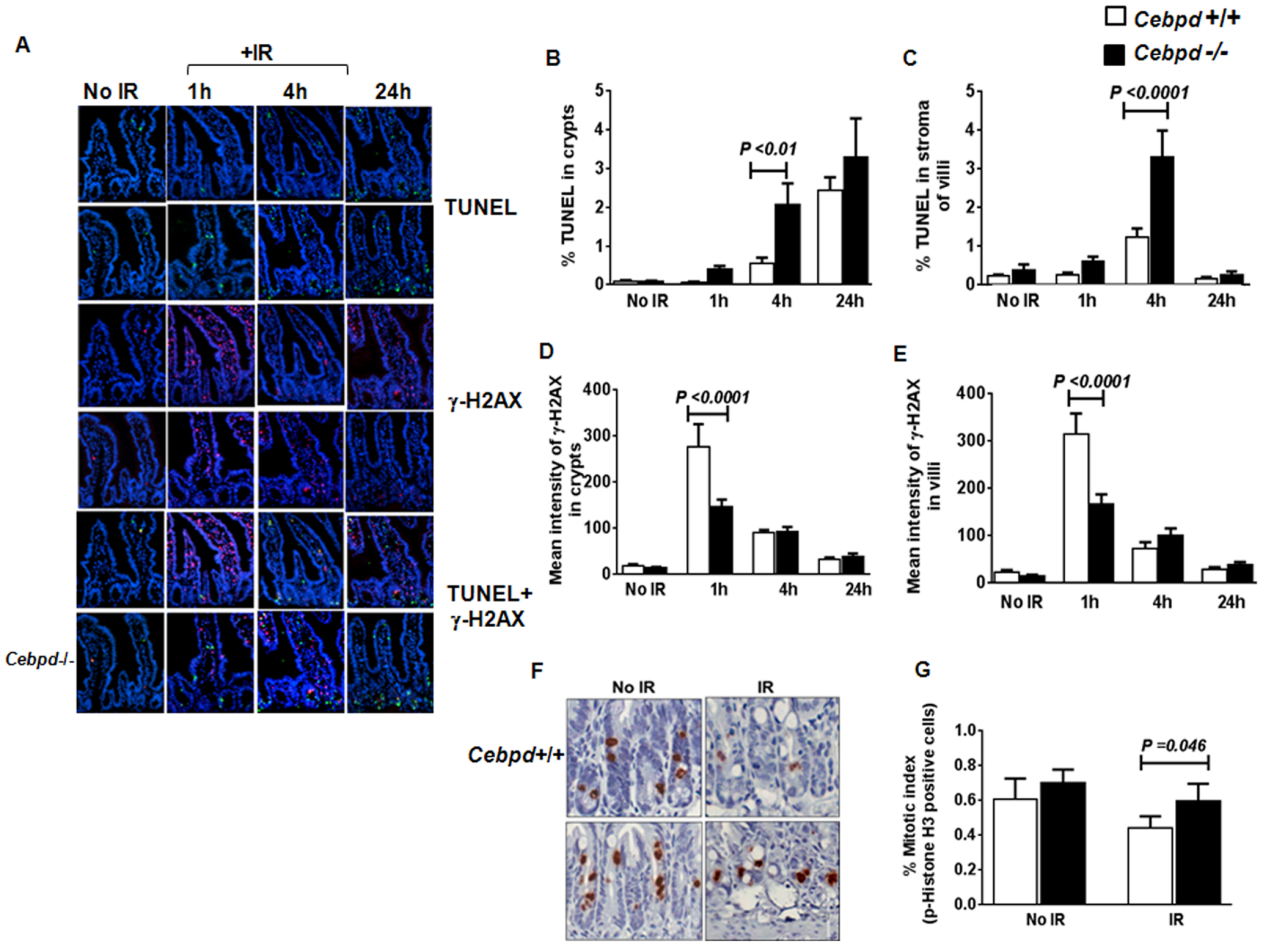
*Cebpd−/−* mice had increased apoptosis and mitotic index and decreased levels of γ-H2AX in intestinal crypts, post-TBI. (A) Representative images of radiation-induced DNA fragmentation (TUNEL, green staining), DNA-damage marker γ -H2AX (red staining), and cellular nuclei (DAPI, blue staining) of proximal jejunums harvested from *Cebpd +/+* and *Cebpd*−/− mice at indicated times after exposure to 7.4 Gy TBI (magnification 10X). (B, C) Quantification of TUNEL-positive cells in intestinal crypts and stromal cells of the villi at indicated time-points after exposure to IR. (D, E) Quantification of γ-H2AX expression levels in intestinal crypts and villi at indicated time-points after exposure to IR. Values are presented as mean ± SEM, n = 4 per genotype per group. (F) Proximal jejunums of *Cebpd+/+* and *Cebpd*−/− mice were harvested at 0 h (No IR) and 24 h (IR) after exposure to 7.4 Gy TBI; representative images of immunohistochemical staining of phospho-histone H3 (Ser28) taken at 40× magnification. (G) Phospho-histone H3 (Ser28)-positive cells across approximately 100 intestinal crypts from unirradiated (No IR) and irradiated (IR) *Cebpd +/+* and *Cebpd*−/− mice were scored and represented as mean ± SEM, n = 4 per group.

Statistical analyses were performed with Graph Pad Prism, version 6.0 (Graph Pad Software). Survival curves were constructed with the Kaplan-Meier method, and 30-day survival rates were compared with the logrank (Mantel-Cox) test in GraphPad Prism (Figure 1). Data are presented as mean ± SEM. Data for Figures 2–7, Figures S1–S2 was analyzed by student's two-tailed *t* test for independent means. A value of *P*<0.05 was considered a significant difference.

## Results

### Increased radiosensitivity of *Cebpd−/−* mice

We investigated the effects of TBI on survival of *Cebpd−/−* and *Cebpd+/+* mice over a period of 30 days. After exposure to 7.4 Gy TBI, *Cebpd−/−* mice died between days 13–15 and showed an overall survival of 28.5%; *Cebpd+/+* mice died on day 16, with 85% overall survival (Figure 1). At 8.5 Gy TBI, *Cebpd−/−* mice died between days 9–13, with 100% mortality; *Cebpd+/+* mice died on days 8–14 with 60% overall survival (Figure 1). Post-irradiation survival of *Cebpd−/−* mice was significantly lower than that of *Cebpd+/+* mice at both doses of TBI. This result demonstrates that C/EBPδ contributes to post-radiation survival.

### Impaired post-irradiation recovery of white blood cells (WBCs), neutrophils, platelets, and myeloid cells of *Cebpd−/−* mice

It has been well established that the lethality induced by these doses of TBI (7.4–8.5 Gy) in mice is primarily attributed to IR-induced hematopoietic tissue damage [2]–[4]. Therefore, we investigated various parameters of hematopoietic injury after a sublethal dose of 6 Gy. At 14 days post-TBI, *Cebpd−/−* mice showed a significant decrease in the absolute numbers of WBCs and neutrophils compared to *Cebpd+/+* mice (Figure 2, A and B). We found a decrease in numbers of monocytes, and lymphocytes in irradiated *Cebpd−/−* mice compared to respective *Cebpd+/+* controls, but this difference was not statistically significant (Figure S1 A and B). There was no difference in the absolute numbers of RBCs before and after irradiation, in both genotypes (Figure S1 C). *Cebpd−/−* mice showed a 3.6-fold decrease in recovery of platelets compared to *Cebpd+/+* mice at 14 days post-irradiation (Figure 2 C), while there was no difference in the unirradiated groups.

Phenotypic analysis of peripheral blood cells revealed no significant difference in B, T, and myeloid cells in unirradiated *Cebpd−/−* mice and *Cebpd+/+* controls (Figure 3 A–C). In the irradiated groups, we found no difference in the recovery of B and T cells, but the post-irradiation recovery of myeloid cells in *Cebpd−/−* mice was significantly reduced compared to *Cebpd+/+* controls (Figure 3 A–D). These data show that C/EBPδ is required for the expansion of the myeloid lineage specifically following TBI.

### Lack of *Cebpd* led to increased radiosensitization of BM-MNCs, and BM progenitors

We next investigated the effects of radiation on counts of BMCs and BM-MNCs in irradiated and unirradiated groups of *Cebpd−/−* and *Cebpd+/+* mice. Although the absolute numbers of BMCs were not significantly different in both genotypes prior to and after TBI (Figure 4 A), BM-MNCs were significantly reduced in *Cebpd−/−* mice after TBI (Figure 4 B). The mononuclear cell population includes HPCs, lymphoid cells (lymphocytes, plasmatic cells), monocytes, and macrophages.

We further investigated whether *Cebpd*-deficient BM progenitor cells were proficient to undergo proliferation and differentiation post-radiation exposure. This was assessed by the ability of BMCs to form colonies 14 days after *Cebpd−/−* and *Cebpd+/+* mice were exposed to a sublethal dose of TBI (6 Gy) or to no irradiation. There were no significant differences between unirradiated *Cebpd−/−* mice and *Cebpd+/+* controls in the ability of the BM progenitors to form colonies (Figure 4 C–E). However, 14 days post-irradiation, the colony forming abilities of BM progenitors from *Cebpd−/−* mice were significantly reduced compared to *Cebpd+/+* controls (Figure 4 C–E). Collectively, these data demonstrate a role for C/EBPδ in the post-radiation proliferation and or differentiation of common myeloid progenitors.

### *Cebpd*-deficiency promotes radiation-induced apoptosis and injury of HPCs and HSCs

It is well established that HPCs and HSCs play predominant roles in radioprotection and are required for short-term and long-term radioprotection, respectively [4], [40]. Therefore, we investigated whether the increased radiosensitivity of *Cebpd−/−* mice was due to decreased survival of HPCs and HSCs *in vivo*. There were fewer surviving multi-potent progenitors/HSCs and HPCs in sublethally irradiated (6 Gy) *Cebpd−/−* mice than in irradiated *Cebpd+/+* mice (Figure 5). *Cebpd +/+* mice retained three times more HPCs and HSCs in their bone marrow than did *Cebpd−/−* mice (Figure 5 A–B). In the unirradiated group, the number of Annexin V-positive cells was not significantly different in both genotypes; however, post-irradiation, the apoptotic ratio of HPCs increased 1.6-fold and that of HSCs increased 1.8-fold in *Cebpd−/−* mice, as compared to *Cebpd+/+* mice (Figure 5 E–F). These results demonstrate that C/EBPδ protects the HPCs and HSCs from radiation-induced damage.

### Increased loss of intestinal crypts and decreased plasma citrulline in *Cebpd−/−* mice post-TBI

Next, we investigated whether GI injury played a role in the lethality observed in *Cebpd−/−* mice. We determined the effects of increasing radiation doses (0, 7.4, 8.5, and 10 Gy) on the survival of intestinal crypts. Intestinal stem cells are quiescent and relatively resistant to radiation; however, after radiation injury (usually 3–4 days post-irradiation), they are stimulated to enter a proliferative phase to repopulate the injured tissue and maintain tissue integrity and function [9], [34], [41], [45]. At 0 Gy, there was no significant difference in the number of crypts in both genotypes, however at day 3.5 post-irradiation, *Cebpd−/−* mice showed a much more rapid decline in crypt survival compared to *Cebpd +/+* mice (Figure 6 A–B). These results indicate that C/EBPδ is necessary to regenerate and repair the IR-induced injury.

Reduced plasma citrulline levels are a widely accepted marker of injury to the intestine. We have previously shown that the post-irradiation reduction in plasma citrulline levels correlates with increased intestinal injury [42]. Without irradiation, plasma citrulline levels of *Cebpd−/−* and *Cebpd+/+* mice were not different; however, at 3.5 days post-8.5 Gy, *Cebpd−/−* mice showed 2.5-fold lower citrulline levels than in *Cebpd+/+* mice (Figure 6 C).

### Increased apoptosis, reduced γ-H2AX expression, and increased mitosis leads to increased intestinal injury in *Cebpd−/−* mice after irradiation

We assessed whether the increased intestinal injury of *Cebpd−/−* mice occurs due to increased apoptosis. In unirradiated tissues, the two genotypes were not different in the amount of apoptosis in the crypts and in stromal cells of the villi; however, in response to radiation, a robust increase in apoptosis was observed between 1 h and 24 h in the intestinal crypts of both genotypes. However, at 4 h post-TBI, the number of TUNEL-positive cells in *Cebpd−/−* mice were 4-fold greater in the crypt epithelium and 3-fold greater in stromal cells of the villi than in respective *Cebpd+/+* controls (Figure 7 A–C). At 4 h post-TBI, positional analysis of TUNEL positive cells revealed significantly increased levels of apoptosis at the +4 position and decreased apoptosis at +8 and +10 positions in crypts of *Cebpd*−/− mice compared to that of *Cebpd*+/+ mice (Figure S2). Although the position of intestinal stem cells has been under intense debate, it has been suggested that intestinal stem cells reside at the +4 position [43]. These results suggest that lack of C/EBPδ induces increased post-radiation damage to the intestinal stem cells in the crypts and to stromal cells of the villi.

By 24 h, apoptosis in intestinal crypts further increased in both genotypes (Figure 7 B) and then declined by day 3.5 (data not shown). In contrast, apoptosis in the stromal compartment returned to basal levels in both genotypes at 24 h (Figure 7 C). At 24 h, post-IR, no significant difference in levels of apoptosis were observed between both the genotypes, whether this occurs due to similar DNA repair capacity in *Cebpd*-deficient and wild type crypts needs further investigation.

γ-H2AX is an indicator of double-strand DNA breaks and is thought to recruit DNA repair proteins to the sites of damage [44]. Next, we compared the γ-H2AX expression at various time-points post-irradiation in both genotypes. In unirradiated mice of both genotypes no difference in γ-H2AX expression was observed. Interestingly, the intestinal crypts of *Cebpd+/+* mice showed a robust 277-fold increase in γ-H2AX expression 1 h post-irradiation, while *Cebpd−/−* mice showed a 146-fold increase (Figure 7 A, D and E). These results suggest that *Cebpd−/−* crypts show an impaired IR-induced DNA-damage response compared to the *Cebpd+/+* crypts and that *Cebpd* may play a role in the initial DNA damage response. Between 4 h and 24 h, γ-H2AX expression declined in both genotypes but remained higher than in the unirradiated groups in the villi and the crypt epithelium. There was no difference in the γ-H2AX expression between the irradiated groups at 4 h and 24 h.

Finally, we investigated whether the decreased expression of γ-H2AX correlates with a lack of cell cycle arrest in intestinal crypt cells of *Cebpd−/−* mice after radiation exposure. The mitotic index of crypt cells was measured by examining the expression of phospho (Ser28)-Histone H3. The mitotic index of intestinal crypt cells was not different in unirradiated mice of the two genotypes (Figure 7 F–G). At 24 h post-irradiation, *Cebpd+/+* mice showed a 2-fold decrease in cells positive for phospho-histone H3, whereas the *Cebpd−/−* mice did not show any decrease and maintained levels comparable to those of unirradiated mice (Figure 7 F). These results indicate that unlike the *Cebpd+/+* crypts, the *Cebpd*−/− crypts do not undergo cell cycle arrest post-irradiation.

## Discussion

Exposure to IR leads to increased oxidative stress, DNA damage, genomic instability and inflammation [2]. C/EBPδ expression is induced by inflammatory signals and its roles in promoting the inflammatory response have been extensively studied [25], [27]. Additionally, C/EBPδ is known to regulate genes involved in oxidative stress, DNA damage, genomic instability [21]–[24]. It can be speculated that C/EBPδ ablation has deleterious effects on the organism that may be due to compromised abilities to modulate IR-induced oxidative stress, repair DNA damage, and/or manage the inflammatory response. This study is the first to examine the role of C/EBPδ in radiation injury. Because lethality after TBI in the 7.4–8.5 Gy dose range results from both hematopoietic and intestinal injury (Figure 1), this study analyzed the effects on bone marrow and intestine in *Cebpd−/−* and *Cebpd+/+* mice.

Radiation generates free radicals and reactive oxygen species that damage vital cellular targets, such as DNA and membranes, and affect the stem cells niches. The bone marrow is extremely vulnerable to cytotoxicity caused by radiation [3]–[8], and injury to the stem cell compartment of the GI tract is the underlying cause of TBI lethality at higher doses [9], [35], [41], [45]. Irradiation, by affecting the bone marrow, has a profound effect on the levels of circulating blood cells, and most of the post-radiation hematopoietic morbidity and mortality is attributed to infection and hemorrhage due to leukopenia and thrombocytopenia [3]–[6]. *Cebpd−/−* mice exhibit higher mortality in response to IR and had significantly fewer WBCs, neutrophils, and platelets than *Cebpd+/+* controls on day 14 post-TBI (Figure 2 A–C). Similar to a recent study in 129S1 mice, we found that C/EBPδ ablation did not affect the differentiated blood cell types such as WBCs, RBCs and platelets under steady state conditions [46]. However, 14 days post-irradiation, while no differences were observed in B and T cells (Figure 3, A–D) and, myeloid cells were significantly reduced in *Cebpd−/−* mice (Figure 3 C and F). This indicates that C/EBPδ plays a critical role in the radiation stress response or “emergency” myelopoiesis. Another family member C/EBPβ has been implicated in “emergency” granulopoiesis in response to infection, thus further demonstrating highly-specific non-redundant functions for this family of transcription factors [47].

While there was no significant difference in the absolute numbers of BMCs before and after exposure to IR (Figure 4 A), BM-MNCs showed a significant reduction after irradiation in *Cebpd−/−* mice (Figure 4 B). A similar radiation-induced decrease in BM-MNCs has been reported for Toll-like receptor 4 (*Tlr4)-*knockout mice [48]. *Cebpd*-deficiency resulted in decreased proliferation of various BM progenitors for the granulocyte, erythrocyte, monocyte, and megakaryocyte lineages (Figure 4 C–E) specifically after irradiation, thus indicating that *Cebpd* plays an essential role in stress-induced proliferation and or differentiation of the common myeloid progenitors. C/EBPδ expression in cells of the myeloid lineage such as granulocytes and neutrophils has been reported earlier [49] and overexpression of C/EBPδ in primary mouse hematopoietic progenitor cells is shown to stimulate myeloid differentiation which is accompanied by upregulation of G-CSF and downregulation of c-myc expression [30], thus it is plausible that C/EBPδ may play a role in post-radiation myeloid differentiation through a similar mechanism.

Although we found no difference in HPCs and HSCs of unirradiated *Cebpd+/+* and *Cebpd*−/− mice, the post-TBI recovery was significantly impaired in *Cebpd−/−* mice implicating C/EBPδ in regulation of survival, renewal, and proliferation of HPCs and HSCs in response to IR (Figure 5 A–D). Moreover, this decrease in recovery of HPCs and HSCs correlated with an increased apoptotic response to radiation (Figure 5 E–F) and with a decrease in BM-MNCs in *Cebpd−/−* mice (Figure 4 B). It is known that, radiation-induced oxidative stress plays a major role in the recovery of HPCs and HSCs [8], [50]. A recent study implicated C/EBPδ in the transcriptional regulation of superoxide dismutase [21]. Therefore, C/EBPδ may protect HPCs and HSCs after radiation via regulation of oxidative stress induced by IR and/or by promoting DNA-damage repair; although this idea needs further validation.

Interestingly, in our studies, exposure to a sublethal dose of TBI (8.5 Gy) led to a dramatic decrease in crypt cell survival in *Cebpd−/−* mice at day 3.5 post-irradiation. The increased mortality to radiation observed in *Cebpd−/−* mice compared to the *Cebpd+/+* mice correlated with decreased survival of intestinal crypts (Figure 6 A–B), increased apoptosis of stromal cells (Figure 7 C) and intestinal stem cells (Figure S2). The increased radiosensitivity of the intestinal stem cells and stromal cells in the *Cebpd−/−* mice suggests that *Cebpd* expression is essential for cell and organismal survival of these cells in response to IR.

Plasma level of citrulline is a well-validated biomarker for functional enterocyte mass [36], [37], [51], and it closely correlates with more conventional markers of intestinal radiation injury, including mucosal surface area and crypt colony survival assays [42]. In *Cebpd−/−* mice, we observed a similar trend of decreased citrulline levels 3.5 days post-TBI (Figure 6 C), again indicative of enterocyte injury.

In response to IR, *Cebpd−/−* mice exhibited increased apoptosis of intestinal stem cells and stromal cells of villi, as compared to *Cebpd+/+* mice (Figure 7 B–C, Figure S2). The increased apoptosis observed in bone marrow and GI tissues of *Cebpd−/−* mice after IR suggests that C/EBPδ may be involved in an anti-apoptotic pathway, such as cell cycle arrest possibly to allow for DNA damage repair processes such as stem cell renewal, proliferation, and differentiation. Consistent with this idea, a recent study has implicated a role for C/EBPδ in anti-apoptotic and anti-inflammatory gene networks in pancreatic β-cells [52].

Exposure to radiation induces double-strand DNA breaks and results in activation of ATM, ATR, and DNA-PKs, which phosphorylate histone H2AX and other substrates (e.g., Chk1, Chk2) to initiate cell cycle arrest until the DNA-damage repair is complete [44]. We found that, while both genotypes robustly induced γ-H2AX expression 1 h post-irradiation, *Cebpd+/+* mice expressed higher levels of γ-H2AX expression in intestinal crypts and villi than the *Cebpd−/−* mice (Figure 7D–E). These results suggest that *Cebpd* may play an important role in the initial stages of DNA damage response but not at later timepoints.

A similar decrease in post-irradiation expression of γ-H2AX observed in mice lacking p53-inducible gene3, Hsp72, Sirt1, p27, and mammalian SWI/SNF [53]–[57] was correlated with an impaired DNA-damage response and an increase in mitosis. Similar to p27-knockout mice, the increased phospho-histone H3 expression in *Cebpd−/−* intestinal crypts compared to that in *Cebpd+/+* mice (Figure 7 F–G) suggests an inability to undergo post-irradiation cell cycle arrest [57]. Collectively, these results are indicative of an impaired DNA-damage response and impaired ability to undergo cell cycle arrest in the *Cebpd−/−* intestinal crypts, which results in increased apoptosis of the intestinal crypts.

C/EBPδ plays an important role in regulation of the inflammatory and stress responses as well as in innate and adaptive immune response [58]–[61], and these functions may also contribute to the increased radiation-induced injury to the intestine and bone marrow of *Cebpd*-deficient mice. The TLR4 receptor plays an important role in inflammatory activation and, similar to the *Cebpd*−/− mice, *Tlr4-* deficient mice are hypersensitive to radiation [48]. A recent study has shown that C/EBPδ and TLR4 engage in a positive regulatory feedback loop and shown that *Cebpd*-deficiency affects the expression of several TLR genes [46]. One can thus speculate that C/EBPδ protects from radiation injury by promoting TLR pathways, which needs to be further investigated.

In conclusion, our studies revealed that injury to the progenitor and stem cells in the hematopoietic compartment and to the stromal and stems cells in the intestine are the likely underlying causes of TBI-induced lethality of *Cebpd−/−* mice. Further studies are needed to elucidate the exact molecular mechanisms of radioprotection which could be mediated via direct or indirect targets of C/EBPδ. This novel protective role of C/EBPδ in radiation injury at the cellular level in normal tissues, will have also have implications in the context of tumors, and needs further investigation.

## Supporting Information

Figure S1 ***Cebpd−*** **/− mice showed a decreasing trend of monocytes and lymphocytes post-TBI.** (A) Monocytes, (B) lymphocytes, and (C) RBCs were counted in unirradiated (No IR) (n = 3/genotype) and irradiated (IR) (n = 3/genotype) *Cebpd*+/+ and *Cebpd*−/− mice 14 days after exposure to 6 Gy TBI. All data are represented as mean ± SEM.
(TIF)

Figure S2 ***Cebpd-*** **deficiency resulted in increased radiation-induced apoptosis in the intestinal stem cell compartment at 4 h post-TBI.** The frequency of TUNEL-positive cells was scored according to cell position. 50–100 half-crypts per animal and 4 animals per genotype were scored to determine the percentage of apoptotic cells. All data are represented as mean ± SEM. *P<0.05.
(TIF)
