# Supplementary figures and images for: C/EBPδ Deficiency Sensitizes Mice to Ionizing Radiation-Induced Hematopoietic and Intestinal Injury
Source: PLoS One. 2014 Apr 18;9(4):e94967. doi: 10.1371/journal.pone.0094967 (PMC3991713; doi:10.1371/journal.pone.0094967)

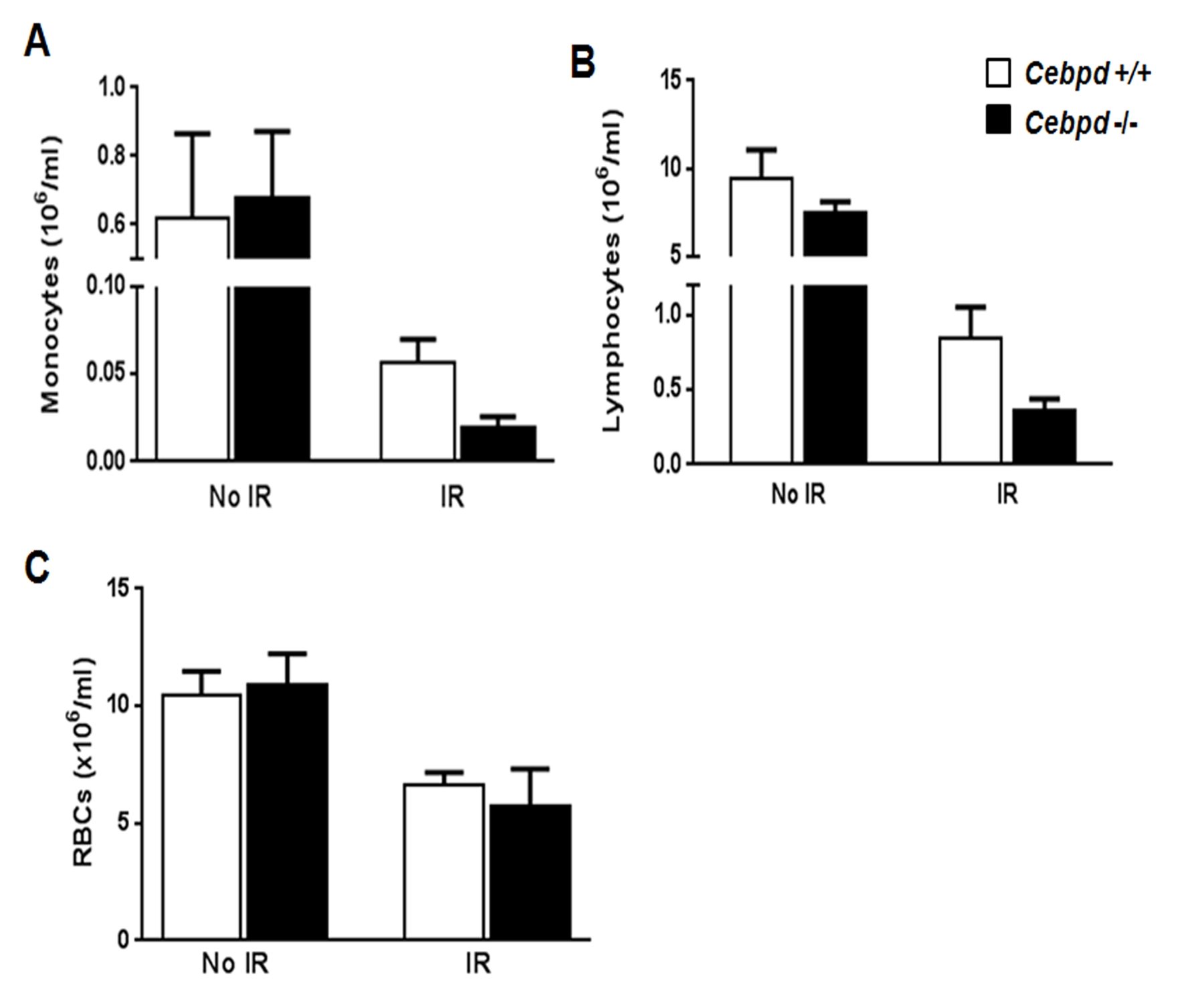

Supplement: Figure S1 — Cebpd− /− mice showed a decreasing trend of monocytes and lymphocytes post-TBI. (A) Monocytes, (B) lymphocytes, and (C) RBCs were counted in unirradiated (No IR) (n = 3/genotype) and irradiated (IR) (n = 3/genotype) Cebpd+/+ and Cebpd−/− mice 14 days after exposure to 6 Gy TBI. All data are represented as mean ± SEM. (TIF) [file pone.0094967.s001.tif]

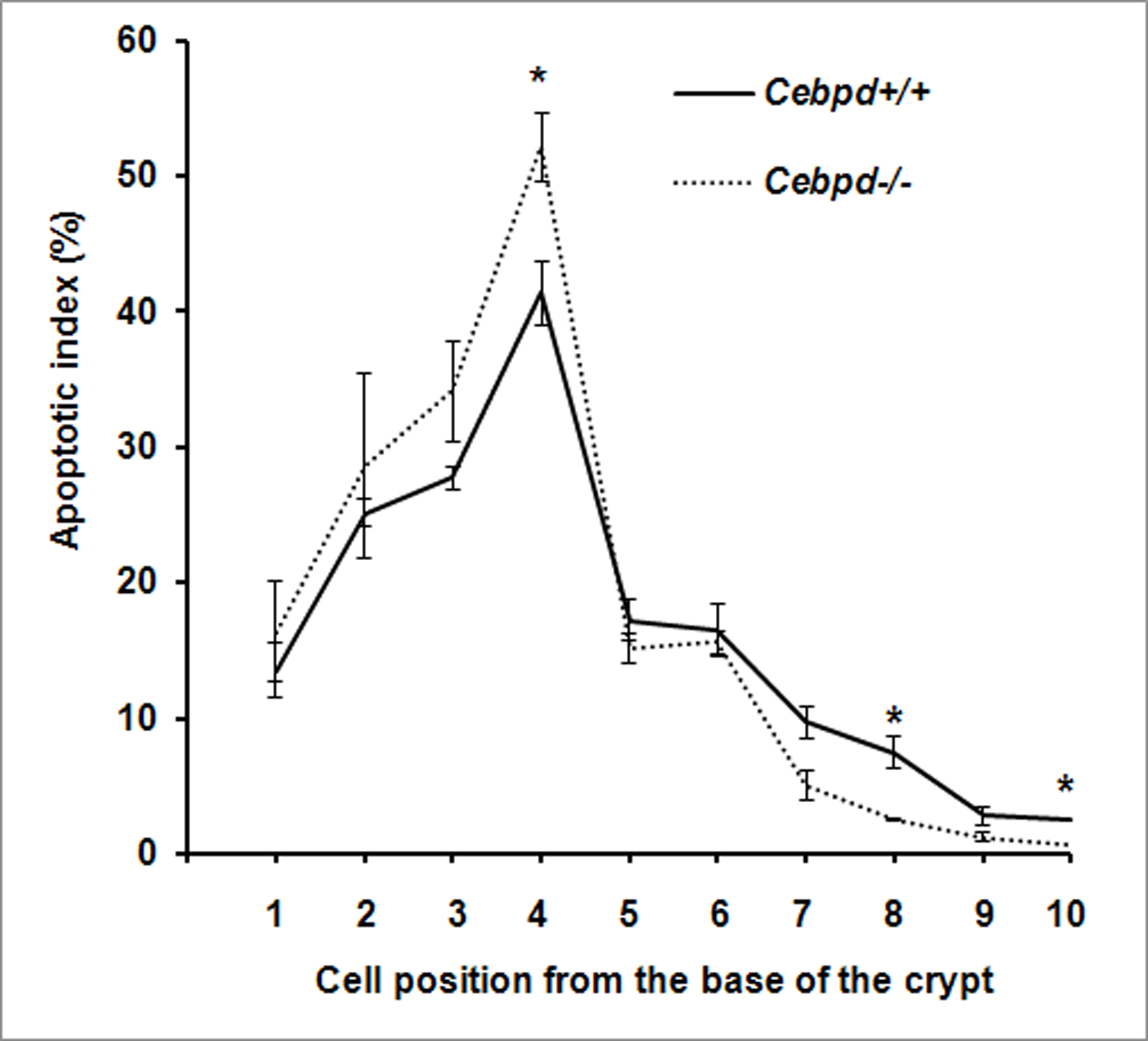

Supplement: Figure S2 — Cebpd- deficiency resulted in increased radiation-induced apoptosis in the intestinal stem cell compartment at 4 h post-TBI. The frequency of TUNEL-positive cells was scored according to cell position. 50–100 half-crypts per animal and 4 animals per genotype were scored to determine the percentage of apoptotic cells. All data are represented as mean ± SEM. *P<0.05. (TIF) [file pone.0094967.s002.tif]
